# The CD133 and CD34 cell types in human umbilical cord blood have the capacity to produce infectious dengue virus particles

**DOI:** 10.1038/s41598-023-37707-8

**Published:** 2023-06-29

**Authors:** Amrita Vats, Tzu-Chuan Ho, Irwin Puc, Chiung-Hsin Chang, Guey-Chuen Perng, Po-Lin Chen

**Affiliations:** 1 Institute of Basic Medical Sciences, College of Medicine, National Cheng Kung University, Tainan, 701401 Taiwan; 2 Department of Microbiology and Immunology, College of Medicine, National Cheng Kung University, Tainan, 701401 Taiwan; 3 Department of Obstetrics and Gynecology, College of Medicine, National Cheng Kung University, Tainan, 701401 Taiwan; 4 Department of Internal Medicine, National Cheng Kung University Hospital, Tainan, 70428 Taiwan

**Keywords:** Cell biology, Immunology, Microbiology, Stem cells

## Abstract

Although dengue virus (DENV) can establish infections in hematopoietic stem progenitor cells (HSPCs), there is little information on dengue virus persistent infection in CD34+ and CD133+ cell surface glycoproteins of hematopoietic stem cells (HSCs). CD34 and CD133 also function as cell–cell adhesion factors, which are present in umbilical cord blood (UCB). In this study, we sought to establish a persistent infection model of DENV infection in UCB using a prolonged period of infection lasting 30 days. Post-infection, the results exhibited a productive and non-productive phase of DENV production. Using a plaque assay, Western blot, and confocal microscopy, we demonstrated that CD133 and CD34 cells are target cells for DENV infection. Moreover, we showed that DENV particles can be recovered from the non-productive phase of DENV-infected CD34 and CD133 cells after co-incubation with Vero cells. We concluded that CD133 and CD34 retain their capacity to produce the infectious virus due to proliferation and their ability to repopulate, as deduced from a BrdU proliferation assay and flow cytometry analysis using t-distributed stochastic neighbor embedding. In summary, the platform to co-culture infected primitive HSCs from their non-productive phase onto Vero cells will give new insights into understanding the DENV dynamics in cell-to-cell transmission and reactivation of the virus.

## Introduction

Dengue virus belongs to the family Flaviviridae and can be antigenically divided, which causes similar symptoms ranging from dengue fever (DF), with mild febrile illness, to life-threatening dengue high hemorrhagic fever (DHF) and dengue shock syndrome (DSS)^1^. Dengue virus contains three structural elements (a capsid (C), membrane (M), and envelope) (E) and seven non-structural elements (NS1, NS2a, NS2b, NS3, NS4a, NS4b, and NS5 proteins)^2^. A recent report from the WHO indicates that the number of dengue cases has increased by more than eightfold over the last two decades, and estimates indicate that there were 4.2 million cases in 2019^3^. Approximately three-quarters of dengue infections are asymptomatic, making this disease difficult to diagnose and treat^4^. Notably, in a report by Duong et al., symptomless people were found to be more likely to spread the infection to mosquitoes and in the community than patients exhibiting clinical characteristics of the dengue profile^5^. Moreover, the propensity of viruses to become undetectable to immune surveillance mechanisms and reactivate under conditions conducive to continuing the reinfection cycle is overwhelming the scientific community and needs more rigorous study^6^. Various evidence indicates that monocytes and macrophages are the primary targets of dengue virus infection and may play a role in dengue transmission^7^. The literature indicates that DENV infects hematopoietic stem cells and alters their proliferative capacity, causing them to produce highly infectious viruses^8–11^. DENV-infected susceptible mammalian cells interact with apoptotic pathways during viral infection pathogenesis. Cells undergoing apoptosis consequently purge the infectious progeny virus from infected cells into the blood stream or cell culture to infect other cells^12,13^. However, cell-free virus propagation is ineffective due to various factors, including variable affinity and multivalent interactions between receptors and viral proteins^14]^.

As DENV replicates in a wide spectrum of cells, the identity of adhesion molecules and receptors that implicate infection depends on the type of host cell and serotype^15^**.** Specifically, the cell to cell transmission of viral RNA has been acknowledged as an alternative route for the spread of progeny virions^13,16–19^. Acknowledging this idea, we employed a platform of co-culture systems to recover the virus from its non-productive phase of infection. Vero cells are commonly used as a co-culture system for improved embryonic development^19–21^. Vero cells, in addition to endothelial cells, are the standard model commonly used in dengue infection. The infectivity of the Vero cell line is higher than that of endothelial cells and slightly lower than that of monocytes^22^. Vero cells are highly susceptible to many viruses in humans and animals, and their membrane proteins are responsible for virus entry.

A previous study demonstrated that stem cells are not easily infected by viruses^23^. However, viruses frequently disturb the survival, differentiation, and proliferation of HSC^24^. It is now common knowledge that a variety of viruses can infect HSC and CD34+ progenitor cells, as shown by various authors, but our understanding of this process remains incomplete^25,26^. To better understand this phenomenon, we examined the susceptibility of CD133 and CD34 cells in UCB towards DENV and their proliferative capacity using a Brdu proliferation assay after prolonged infection.

Human and other mammalian cells are usually infected by DENV through endocytosis mediated by receptor(s), including dendritic cell-specific ICAM-grabbing non-integrin DC-SIGN, mannose, and C-type lectin domain containing 5A(CLEC5A)^27^. There is no distinct hypothesis that explains the entry of DENV onto the surface of primitive hematopoietic stem cells^28^. There is also no evidence or studies on observed receptor-mediated entry into clathrin-coated pits in stem cells. The literature suggests the direct infection of hematopoietic progenitor cells^11^. In the present study, we showed that DENV infection can persist in UCB with an alternating phase of virus production and dormancy. The virus can propagate in the CD133+ and CD34+ cells of UCB and does not disrupt the cell surface expression of CD133 and CD34 to a great extent. The exposure of Vero cells to DENV-infected cells from the non-productive phase of the UCB carrier culture supports the propagation of CD133+ and CD34+ cells and induces cytopathology. The use of a co-culture permits intercellular contact while maintaining the pure cell population of stem cells and purging the virus. Our data establishes that co-culturing dormant infected cells using Vero cells is a robust platform for scalable virus production. This study is crucial in determining that the presence of another cell population or heterologous cells may improve the infection process through cell behavior.

## Materials and methods

### Study design for in-vitro DENV infection of UCB cells and ethics declaration

All enrolled pregnant women signed an informed consent form for UCB collection based on the Helsinki declaration, and the study was approved by the ethics committee of the National Cheng Kung University Hospital in accordance with the protocol (IRB, A-ER-103-184) approved by the Institutional Review Board. UCB samples were prepared for the experiment using an RBC lysis buffer to remove red blood cells and obtain mononuclear cells. To establish persistent infection, 1 × 10^6^ cells/ml cells were counted and infected with DENV-2 (16881) at a MOI = 1 of the virus; the cells were maintained in this state for 12 h. Cells were then centrifuged at 400 rcf for 8 min.

The pellet was dissolved in 10% RPMI and cultured in a 25-cc culture flask for 3 h. After 3 h, only suspension cells were collected and transferred to flow tubes. The Supernatants were harvested at different days post-infection (p.i) (Days 2, 5, 10, 20, 25, and 30) to quantify the virus titers through a traditional plaque assay technique^29^. An aliquot of infectious supernatant was periodically removed at the indicated time points and assayed for infectious virus titer, but we ensured that an equal amount of fresh culture medium was added back to maintain the initial volume. The effect of GM-CSF on DENV-infected UCB was investigated by adding GM-CSF (PeproTech, Rocky Hill, NJ, USA) into the culture medium without cytokines (control) under an optimized concentration of 0.1 ng/ml of GM-CSF (test) on days 25 and 30.

### RNA extraction and real time PCR for DENV-infected UCB supernatant

Total RNA was extracted from the DENV-infected UCB supernatant collected at various harvest time points (Days 2, 5, 10, 20, 25, and 30). The RNA was isolated using Invitrogen and a Pure link Viral RNA/DNA mini-isolation kit according to the manufacturer’s instructions (Invitrogen, Carlsbad, CA, USA). In total, 5 µg of RNA was converted into cDNA using a super script III first-strand synthesis system for RT-PCR with random hexamers (Invitrogen, Carlsbad, CA, USA). The concentration of cDNA was quantified using a nanodrop (Thermo Scientific, MA, USA). A template of 1 µg cDNA was adjusted to carry out real time PCR using a SYBR green master mix reagent containing 0.8 µg of forward primer (5′TGGCTGGTGCACAGACAATGGTT-3′) and reverse primer (5′GCTGTGTCACCCAGAATGCCAT-3′)^30^, including 10 µl of 2xq PCRBIO SYBR green blue mix. The thermal profile for optimizing real time PCR was as follows: 1 cycle of denaturation at 95 °C for 2 min, followed by 40 cycles of amplification at 95 °C for 5 s and 60 °C for 30 s using a thermal cycler. A standard curve was also prepared using a serial dilution of amplified DENV (DV2) stock from Vero-infected cells to a concentration of 1 × 10^1^ to 1 × 10^6^ copies/ml.

### Cells and viruses for co-culture

In a 2 cm dish of DEMEM containing 5%FBS, we seeded 1 × 10^5^ Vero cells. Once the cells reached confluence, the media were removed, and donor cells from DENV-infected CD133+ and CD34+ UCB cells harvested on day 25 p.i. were placed onto the monolayer of Vero cells (5% CO_2_, 37 °C) and allowed to co-cultivate for 7 days to observe the cytopathic changes. The cells were reconstituted with 500 μl of 2% DMEM. Subsequently, two hundred microliters of the supernatant were collected on days 2, 5, and 7 post-co-culture, and each well was replenished with an equal amount of 2% FBS DMEM. The co-cultured supernatant and cells were administered to quantify the viral load and analyze the percentage of gated cells of the surface markers CD133 and CD34 using BD LSR Fortessa (BD Biosciences, Franklin Lakes, NJ, USA).

### Flow cytometry analysis of DENV-infected UCB and co-cultured Vero cells

DENV-infected UCB cells were collected from the different time points (Days 2, 2, 5, 7, 10, 20, 25, and 30) and immune-stained with stem-cell-mediated CD34-FITC mouse anti-human and CD133-PE mouse anti-human antibodies, with the respective isotypes of FITC-mouse IgG1κ and PE-mouse IgG1κ (BD Biosciences, Franklin Lakes, NJ, USA). A similar staining protocol was followed when analyzing the presence of CD34 and CD133 in co-cultured cells after day 7 of infection. The cells were trypsinized using 0.25% trypsin and incubated for 5 min at 37 °C. Cells were then collected and centrifuged at 250 rcf for 6 min. The harvested cells were stained with PBS containing 1% BSA and mouse anti-human surface-marker antibodies specific for CD34 FITC IgG1 and CD133 PE IgG1 and conjugated with their respective isotype controls (obtained from BD Biosciences). After staining for 30 min with surface-marker antibodies on ice, the cells were washed with PBS containing 0.1% BSA. Cell data were acquired using an LSR Fortessa (BD Biosciences, Franklin Lakes, NJ, USA) for flow cytometry analysis, and the gated cells were analyzed using the Kaluza software V7 (Beckman Coulter, Brea, CA, USA).

### Mapping of CD133, CD34, and NS1 using t-distributed stochastic neighbor embedding

To observe the cell distribution from d1-d30, a fast t-SNE was run after concatenation from 5 replicates of UCB with relevant markers CD133 ± NS1 ± and CD34 ± NS1 ± and the sample ID. The parameters were set as iterations = 1000 and perplexity = 50 to depict the contours of cell distribution and density in DENV-infected UCB cells using Flow Jo V10 (BD Biosciences, Franklin Lakes, NJ, USA).

### Confocal capture imaging

Immunofluorescence staining was performed to demonstrate the association of NS1 with CD133 and CD34 in UCB. After blocking in 1% BSA, the cells were incubated with primary antibodies CD34-FITC and CD133-PE (BD Biosciences, Franklin Lakes, NJ, USA). Then, the cells were fixed with 4% paraformaldehyde and attached to slides using a cytospin (Thermo Scientific, MA, USA) at 400 rpm for 8 min. The monolayer was washed twice in PBS and permeabilized (0.3% Triton X-100 with 1% BSA in PBS) and finally washed twice again in PBS. The cells were labeled using NS-1 rabbit polyclonal antibody (Gentex, Zeeland, MI, USA) in a permeabilization buffer at 4 °C for 2 h and washed twice again in PBS. The cells were further incubated with AF-488-donkey anti-rabbit (Invitrogen, Carlsbad, CA, USA) and AF568-goat anti-rabbit (Life Technologies, Carlsbad, CA, USA) secondary antibodies for 1–2 h in the dark at room temperature and covered with the antifade reagent DAPI. Fluorescent images were captured in an Inverted Confocal Microscope FV-1000 (Olympus, Shinjuku City, TYO, Japan).

### Magnetic bead sorting of CD133 and CD34 from UCB

To isolate the pure fraction of CD133 and CD34, cells were labeled with their respective antibodies and incubated using a MACS buffer for 30 min with intermittent shaking every 10 min. The cells were separated using a magnetic column following the manufacturer’s instructions (Miltenyi Biotech, Bergisch Gladbach, Germany) and harvested at different time points (Days 2, 3, 5, 7, 10, 14, 20, 25, and 30). To ensure that we achieved a pure population of CD34 and CD133, we resuspended the positive fraction of CD34^+^ and CD133^+^ in a MACS buffer to elute the more enriched population of CD34^+^ and CD133^+^ from the mixture of the negative fraction of the CD34^−^, CD133^−^, and CD133^+^ populations by repeating the magnetic bead sorting twice. In the supplementary section, we report the purity of the gating percentage of CD34^+^ and CD133^+^ cells obtained after magnetic bead sorting using flow cytometry from one of the representative HUCB donors (Supplementary Fig. S8).

### Western blot analysis

Vero cells were co-incubated with DENV-infected specifically sorted CD133+ and CD34+ cells. The infected cells were incubated at 37 °C for up to 7 days. After incubation, the cells were lysed with 2% sodium dodecyl sulphate (SDS), and the lysates were denatured by boiling for 15 min. Denatured DENV-infected co-cultured cells were electrophoresed in a 12% polyacrylamide gel and blotted onto a nitrocellulose membrane with a 0.45 µm pore size (Bio-Rad laboratories, Hercules, VA, USA). The membrane was cut into strips and blocked with 5% skimmed milk in PBS for 45 min at room temperature (RT). Polyclonal rabbit antibodies directed against structural proteins of dengue virus NS-1, E protein, capsids, and PrM protein were diluted in 5 ml of 5% skimmed milk (blocking buffer) at a dilution of 1:2000 and incubated overnight at 4 °C. On the following day, the strip was washed three times with 5-min intervals between washes using PBS-Tween 20. Next, 5 ml of 1:4000 diluted goat anti-rabbit IgG peroxidase was added and incubated for 1 h at RT. The washing steps were repeated as already described above. Chemiluminescent substrate reagent A and B (T-Pro lumi fast plus chemiluminescent substrate) were added and mixed, followed by incubating the strip for 1 min at RT (T Pro-Biotechnology, New Taipei, Taiwan R.O.C).A total of 3 replicates were performed to confirm the expression of protein.

### Brdu proliferation assay

To evaluate the proliferation of CD133 and CD34 in DENV-infected UCB, Brdu was incorporated into the cells on days 25 and 30, and the cells were harvested on days 27 and 32. Subsequently, the cells were stained with fluorochrome-conjugated surface-marker primary antibody CD133-PE and CD34-PEcy7 (BD Biosciences, Franklin Lakes, NJ, USA) in an appropriate volume of staining buffer. The cells were then incubated for 30 min at 4 °C in complete darkness. After washing with a staining buffer, the cells were fixed and permeabilized with a saponin buffer. Then, the cells were resuspended in 100 µl containing DNase1 and incubated for 1 h at 37 °C. After washing the cells with a flow cytometry staining buffer, they were stained with anti-Brdu Ab-FITC (BD Biosciences, Franklin Lakes, NJ, USA), mixed, and incubated for 20–30 min at RT.

### Institutional review board statement

Pregnant women with written informed consent participated in this study, following the protocol (IRB, A-ER-103-184) approved by the Institutional Review Board of National Cheng Kung University Hospital.

### Informed consent statement

Pregnant female participants were enrolled in this study according to the ethical guidelines of the National Cheng Kung University Hospital, Department of Obstetrics and Gynecology.

## Results

### DENV viral load and infectivity in UCB using a plaque assay and real time PCR

UCB cells were infected up to day 30 to quantify their virus production in pfu/ml and their CT values in the DENV-infected supernatant (Table 1). RT-PCR was used as a proxy for probable infectious virus in a UCB culture supernatant. We used an initial viral inoculum of 1MOI on 1 × 10^6^ cells to verify the plaque titer. Our results demonstrated low, medium, and high DENV infection in the UCB. The plaque assay titer showed a high viral load (pfu/ml) up to day 5, whereas in UCBs on days 20 and 25 for donors 1 and 2, there was no detectable viral load. Viral load (pfu/ml) could also be observed on day 30, signifying possible reactivation of the virus.

**Table 1:** DENV viral load and infectivity in UCB using a plaque assay and real time PCR. Virus production during the prolonged DENV infection process in UCB for 5 donors. CT value and viral load (pfu/ml) in DENV-infected UCB supernatants

Days P.I	Donor 1		Donor 2		Donor 3		Donor 4		Donor 5	
	Pfu/ml	CT value	Pfu/ml	CT value	Pfu/ml	CT value	Pfu/ml	CT value	Pfu/ml	CT value
2	8750	26.6	1250	33.55	0	38	138	38	12.5	38
5	250	24.34	4375	29.58	12.5	33	138	33.90	25	38
10	4875	31.54	1250	31.98	375	35.8	13	(−)	0	(−)
20	0	30.96	0	27.43	100	21	75	(−)	0	(−)
25	50	38	12.5	27.13	163	36.24	0	29	50	(−)
30	125	24.49	1250	35.92	50	20.02	25	26.51	125	38

*CT value* cycle threshold, *pfu/ml* plaque forming unit, (−) undetectable.

We observed that the pfu/ml value during infection was incredibly low over time. We calculated the arithmetic and geometric mean of the ct value to estimate the detection limit of RT-PCR. During the first day post infection, the geometric mean and arithmetic mean was (34.5, 34.8, CI 95% 28–41). On day 5, the geometric mean was (31.4, 31.76, CI 95% 25–38). On day 10, the geometric mean was (33, 19.86, CI 95% − 2.7 to 42). On the following days 20, 25, and 30, GM and AM were evaluated as (26.1, 15.8), (32.2, 26.0), and (28.17, 28.9) with a CI 95% of (− 2.6 to 34.4), (7–45), and (19.4–38), respectively. To achieve statistical significance, we used two-way Anova statistical analysis, and a Wilcoxon matched pair signed rank test with a non-parametric paired t-test. There was a difference in the detectable CT value between the different days after infection (p = 0.037). The readouts from the RT-PCR CT value and infectious virus titers were not concurrent. The detection of RE showed variability in the detection of pfu/ml, and the CT value varied over time, making it difficult to interpret the co-relationship between Q-PCR and virus titers in certain donors and at certain time points.

This result could be due to the lower detection limit of the CT value for sensing viral RNA due to factors related to the stability of RNA and storage. However, the presence of a high CT value > 35 in some replicates of UCB did not rule out the presence of infectious RNA virus because the virus was recovered from these UCB donors with a CT value of > 35 and later obtained through a co-culture. Moreover RT-PCR detected viral nucleic acid but not the infectious capability of the virus particles.

Total RNA was quantified in supernatant from the donors and tabulated in Table 1 to compare against the pfu/ml values from the plaque assay. To validate and calibrate our Q-PCR data, in parallel, we also quantify the viral titers of amplified DENV supernatant virus stock from Vero cells. A standard RNA curve was generated measured from a log dilution of 10^1^, 10^2^, 10^3^, 10^4^, 10^5^, and 10^6^. The mean CT values (n = 5) obtained in the corresponding dilutions were plotted against the corresponding serial dilutions (Supplementary Fig. S1).

### Production of infectious virus from CD133+ and CD34+ infected cells

A series of experiments was carried out to determine the infectious virus titers in CD133 and CD34 sorted cells in the DENV-infected UCB supernatant. In the first step, we determined that CD133 and CD34 could achieve a noteworthy trend of viral replication in specific sorted cells of CD133+ and CD34+. After 14 days of infection, UCB replicates 1–3 did not produce infectious virus particles in the sorted cells of CD133+ and CD34+. Replicate number 4 was able to maintain virus production until day 30 (Table 2).

**Table 2:** Production of infectious virus from CD133^+^ and CD34^+^ infected cells. Virus production during a prolonged DENV infection process lasting up to 30 days.

Time of harvest (D1-D30)—Viral load (pfu/ml)										
Cell type	UCB	Days P. I								
	Donors	2	3	5	7	10	14	20	25	30
CD133+	1	0	250	100	0	150	0	0	0	0
	2	175	50	100	50	150	275	0	0	0
	3	0	75	163	250	0	0	0	0	0
	4	125	186,250	383,750	206,250	9875	7125	0	12.5	12.5
CD34+	1	125	50	150	100	0	0	0	0	0
	2	100	150	275	50	0	0	0	0	0
	3	0	75	63	0	250	0	0	0	0
	4	0	0	17,500	15,000	100,000	0	38	1000	38

**Figure 1:**
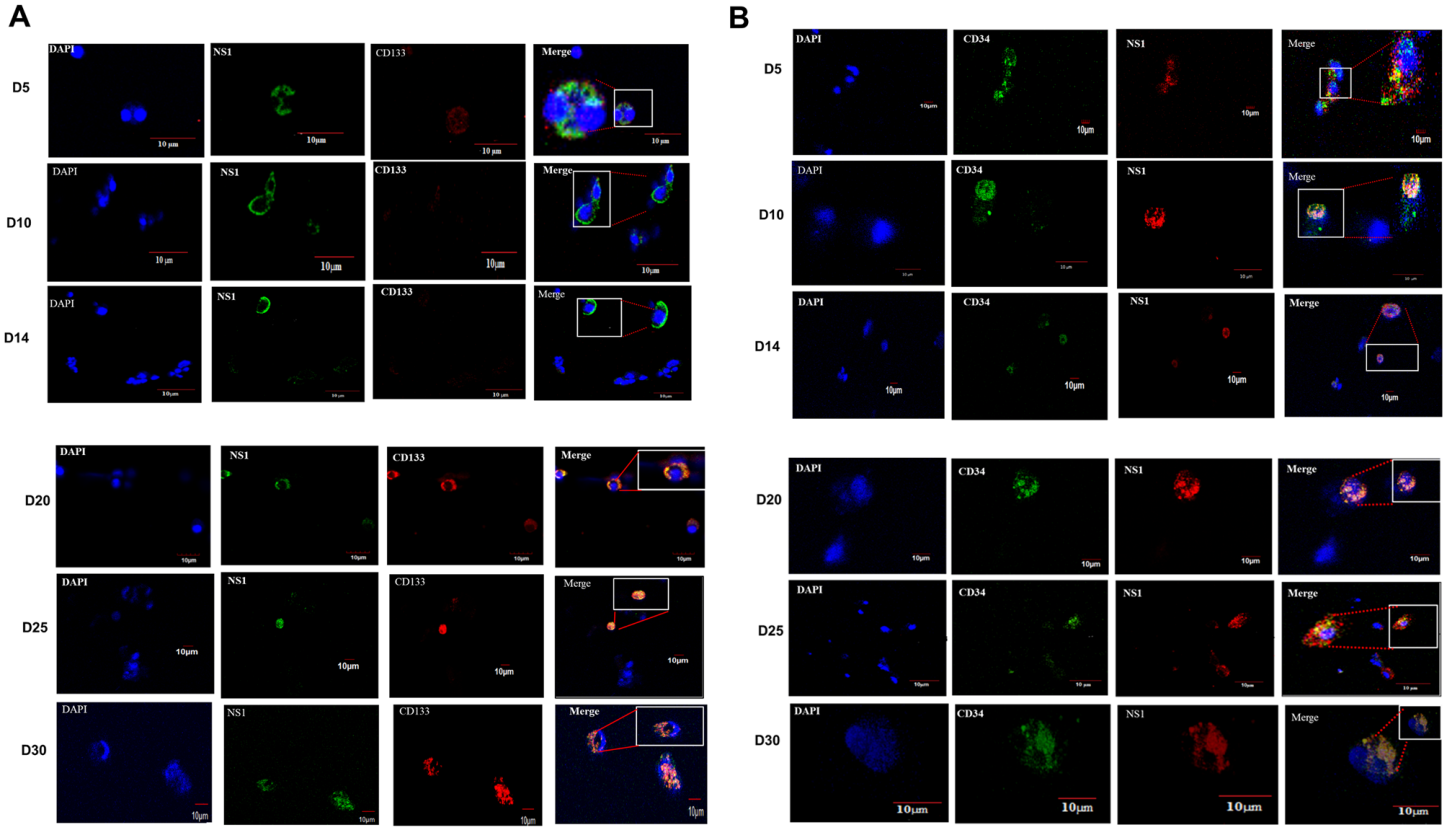
Confocal microscopy showed that the DENV non-structural protein NS1 was associated with CD133 and CD34. (**A**) The non-structural protein NS-1 was observed in the early phase of infection with no CD133 signal. The protein level of CD133 was attenuated, suggesting that CD133 cells resulted in cell death due to high virus production. On day 5, a small dot of CD133 was present around the periphery of NS1 infected cells. From days 21 to 30, NS1 was largely co-localized with CD133, suggesting that they were segregated prior to latency. (**B**) Upregulation of CD34 appeared as red orange spots with complete colocalization with CD34. This result indicates that CD34+ cells predominantly had an intimate association with NS1.

 Our next objective was to determine the association of CD34 and CD133 with NS1 after prolonged infection at various time points (days 5, 10, 14, 21, 25, and 30). Confocal microscopy revealed the aggregation of complete puncta of CD34 and CD133 with NS1 on days 21, 25, and 30. Surprisingly, the formation of puncta with CD133 on day 5 was dispersed. The expression of CD133 on day 10 was not discernible without loss of the NS1 signal. Following prolonged infection, CD133 and NS1 presented a complete association indicating that the CD133-containing microdomain could have been segregated and carried during early infection, thus maintaining the stem cells’ properties. However, CD34 did not deliver such a pattern. Upregulation of CD34 indicates that CD34^+^ cells were predominantly associated with NS1 (Fig. 1). We concluded that DENV NS1 secreted from cells during the initial phase of infection interacted with a pool of CD133 and CD34 cells and remained co-localized.

#### Recurrence of virus from the unproductive phase of DENV-infected UCB after co-incubation with Vero cells

**Figure 2:**
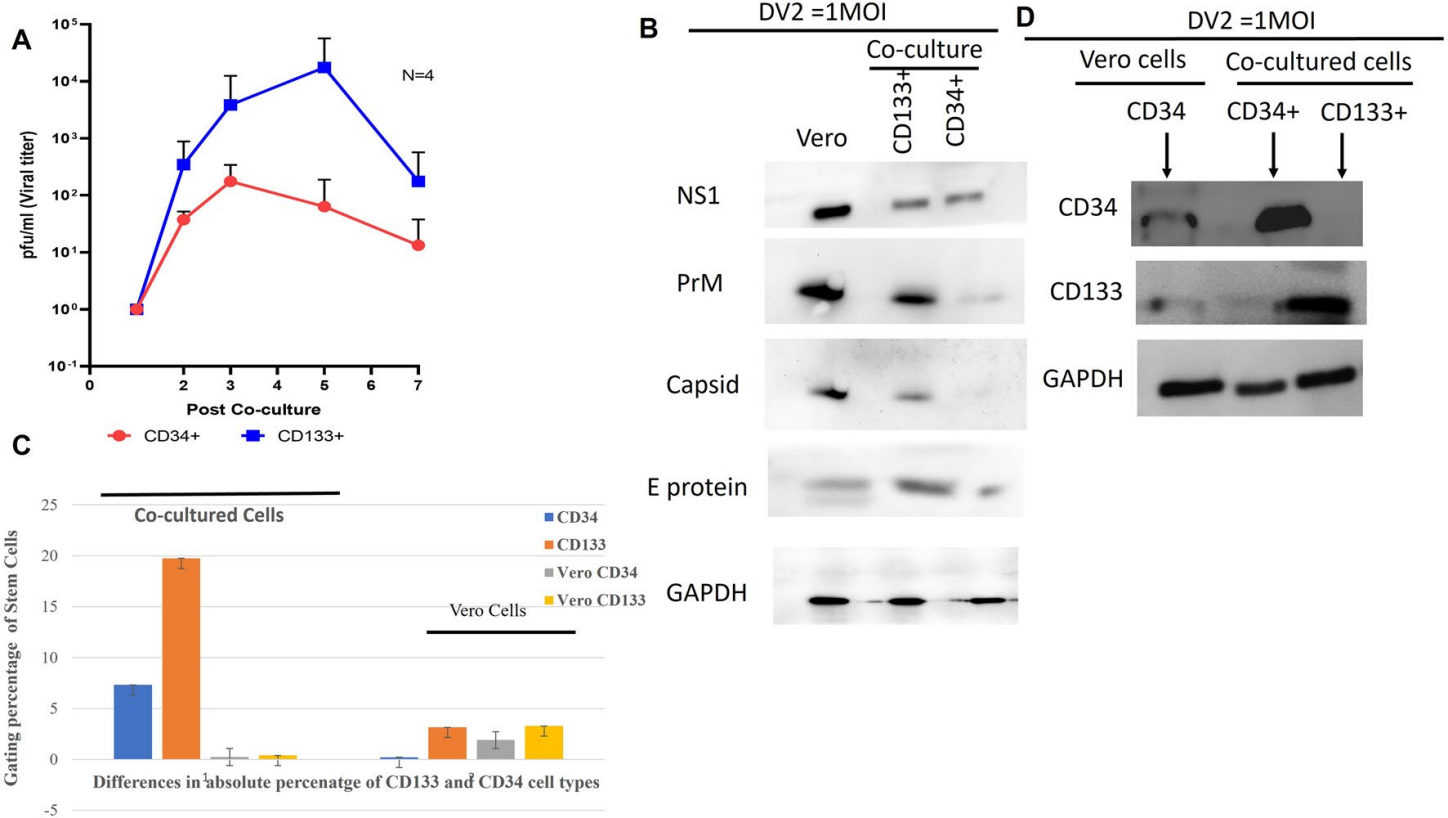
Efficient recovery of virus from the non-productive phase of infected cells. Phenotypic and functional characterization of CD133 and CD34 in co-cultured cells and Vero cells. (**A**) Virus production in the post-co-culture of CD133 and CD34 cells sorted from the carrier culture of UCB. Co-cultured on day 25 (n = 4). Related to Table 3. (**B**–**D**) Functional characterization of stem-cell-mediated antibodies and DENV protein. (**B**) The expression of DENV protein in co-cultured cells indicates that DENV protein is transmitted via latent infected cells to naïve Vero cells. (**C**) Comparison of the percentage of gated cells in naïve Vero cells and cells after being co-cultured using multicolor flow cytometry. (**D**) Detection of CD133 and CD34 protein in co-cultured cells indicates that infected cells propagated on Vero cells. Internal controls derived from DENV-infected Vero cell lysate were used in parallel. A representative image from total of 3 replicates of western blot. Original blots are presented in Supplementary Fig. S6A–D.

The carrier culture with DENV-infected UCBs from the inactive phase of infection was co-cultured with Vero cells. We uncovered virus recovery from the specific sorted cells of CD133^+^ and CD34^+^. The CD133^+^ titer peaked on day 5 but decreased onward until day 7 (Fig. 2A). DENV-infected UCBCs with undetectable virus in the supernatants on day 25 were collected and assessed for infection using a co-culture. These cells were also frozen and thawed to facilitate complete release of the viral progeny. Surprisingly, CD133^+^ produced more viruses after co-culturing compared to CD34^+^ cells (Table 3).

**Table 3:** Reactivation of virus from the unproductive phase of DENV-infected UCB after co-incubation with Vero cells. DENV-infected UCBCs with undetectable virus in the supernatants on day 25 were collected and assessed for infection using a co-culture.

Co-cultured cells (Day of co-culture, day 25)	Days harvested after co-culturing with a viral load (pfu/ml)					
	UCB	Days P. I				
Cell type	Donors		2	3	5	7
CD133+	1		125	12.5	0	0
	2		125	50	0	0
	3		50	0	0	0
	4		1300	19,250	8750	875
CD34+	1		50	250	0	0
	2		25	25	250	50
	3		50	375	0	0
	4		25	50	0	0

#### Detection of DENV protein in co-cultured cells indicates infected cells transmitted the DENV particles

Antibodies against different protein components of DENV-infected sorted stem cells of CD133 and CD34 were detected in co-cultured cell lysate. Antibodies against NS1, E, capsid, and PrM were detected in CD133+ and CD34+ co-cultured cells (Fig. 2B**).** The expression of NS-1 was high and similar in CD133+ and CD34+ co-cultured cells. Nonetheless, PrM expression was weak and did not have detectable levels of capsid protein in CD34+ co-cultured cells in comparison to CD133+ co-cultured cells. These results suggest that DENV-infected CD133 retains antibodies towards NS1, capsids, and PrM proteins. However, CD34+ did not sustain capsids or PrM after harvesting the co-cultured cells on day 7, which coincided with a decrease in the peak viral titers of CD34+ and CD133+ co-cultured cells, as observed in Fig. 2A. Several replicates of western blot FOR DENV mediated antibodies were performed from the co-cultured setting. Considering the fact, the E protein expression is not very strong presented in Fig. 2B. We have presented another replicate of the blot of E protein expression in co-cultured cells of CD34, CD133 in Supplementary Fig. 6B. The original images are presented in the supplementary section.

#### Functional characterization to show the influence of CD133 and CD34 in co-cultured cells and Vero cells

After coculturing infected CD133+ and CD34+ from the carrier culture, and to ensure that the virulence of DENV-infected UCB onto Vero cells was due to CD133 and CD34, we used flow cytometry and Western blot to examine the relative phenotypic expression of CD133 and CD34 in Vero cells. Flow cytometry analysis based on the percentages of CD133 and CD34 showed that CD133 was indeed propagated onto Vero cells after co-culture. However, CD34 expression was lower than that of CD133 **(**Fig. 2C**)**. We detected the protein expression of CD133 and CD34 in co-cultured cells with Western blot strips derived from Vero cell lysate and CD133 and CD34 co-cultured cells. Functional characterization of CD133 and CD34 using a Western blot revealed that signal strength was strongest in co-cultured cells **(**Fig. 2D**)**. To strongly confirm the expression of CD133 in DENV infected co-cultured cells, we have presented an additional blot of CD133 expression in Supplementary Fig. 6C from another replicate of the experiment.

#### DENV infection does not impair the surface expression of CD133 and CD34 without loss of NS1

**Figure 3:**
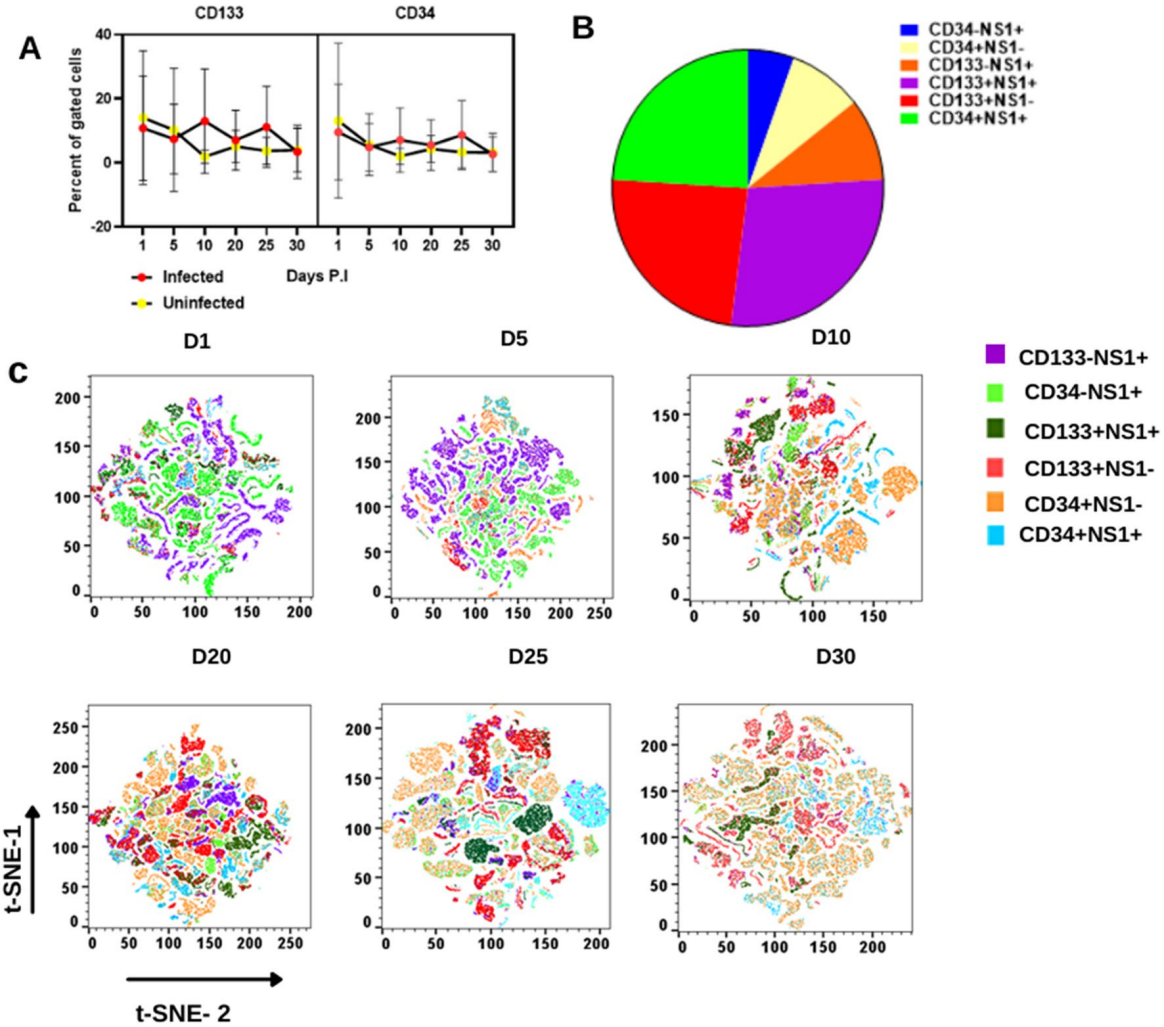
HSC properties were not impaired by long-term infection. (**A**) Expression of CD133 and CD34 representing the percentage of gated cells of CD133 and CD34 in infected and uninfected UCB cells. (**B**) Average phenotypic frequency of CD34+/−/NS1+/−CD133+/−NS1+/− to show the quantitative comparison of each subset. (**C**) Topological view of CD34+/−/NS1+/−CD133+/−NS1+/− after the effects of DENV infection in UCB using multicolor flow cytometry analysis and analyzing the gated cells using t-SNE in Flow Jo V10. Concatenation from 5 replicates of UCB revealed that the percentage of gated cells was explicitly defined using a contour plot to delineate the contour map of each subset of CD34+/−, CD133+/−/NS1 + using color-specific color intensity. See also Supplementary Fig. S2.

Based on the above outcomes of DENV infection in CD133 and CD34 cells and the ability of these cell types to recover the virus after its non-productive phase, it is imperative to understand the function and regulation of CD133 and CD34 cells. Flow cytometric analysis showed that after 4 weeks of infection, CD133 and CD34 gating percentages were higher at a certain time point but decreased on days 5, 20, and 30 in DENV-infected cells in comparison to uninfected UCBs (Fig. 3A). The difference was non-significant. Figure 3B depicts the average phenotypic frequency of CD34 ± /NS1 ± CD133 ± NS1 ± CD133 ± NS1 ± CD133 ± NS1 ± in demonstrating a quantitative comparison of each subset. A bar diagram indicating the frequency of CD34 + NS1, CD133 + NS1, and CD133 + NS1 + cells is presented in Supplementary Fig. S3. The increase in cell number was significant.

To further explore the different subsets, t-SNE plots were drawn to define each subset with distinct multiple spatial contours, and all the subsets were visually distinguished and partitioned using a contour plot (Fig. 3C). Most of the cells were CD133-NS1+ and CD34-NS1+ at the beginning of days 1 and 5. The CD133-NS1+ population, on the other hand, showed an increase in expression from day 10 to 30. For CD34 + NS1 + populations, expression fluctuated as the days of infection progressed, exhibiting decreased expression level on days 25 and 30 compared to CD133-NS1+. This result reflects CD133’s heterogeneity, indicating that CD133 reverted to its original phenotype when attenuated at various stages of infection; this result could also indicate the state of differentiation of CD133 and CD34 because of the indirect effect caused by DENV infection. Overall, the results suggest that DENV does not impair the function of CD133 and CD34, is not killed due to infection, and accounts for the permissive subsets of DENV infection.

#### DENV infection perturbed the cell proliferation of CD133 and CD34 after prolonged infection and contributed to viral load

**Figure 4:**
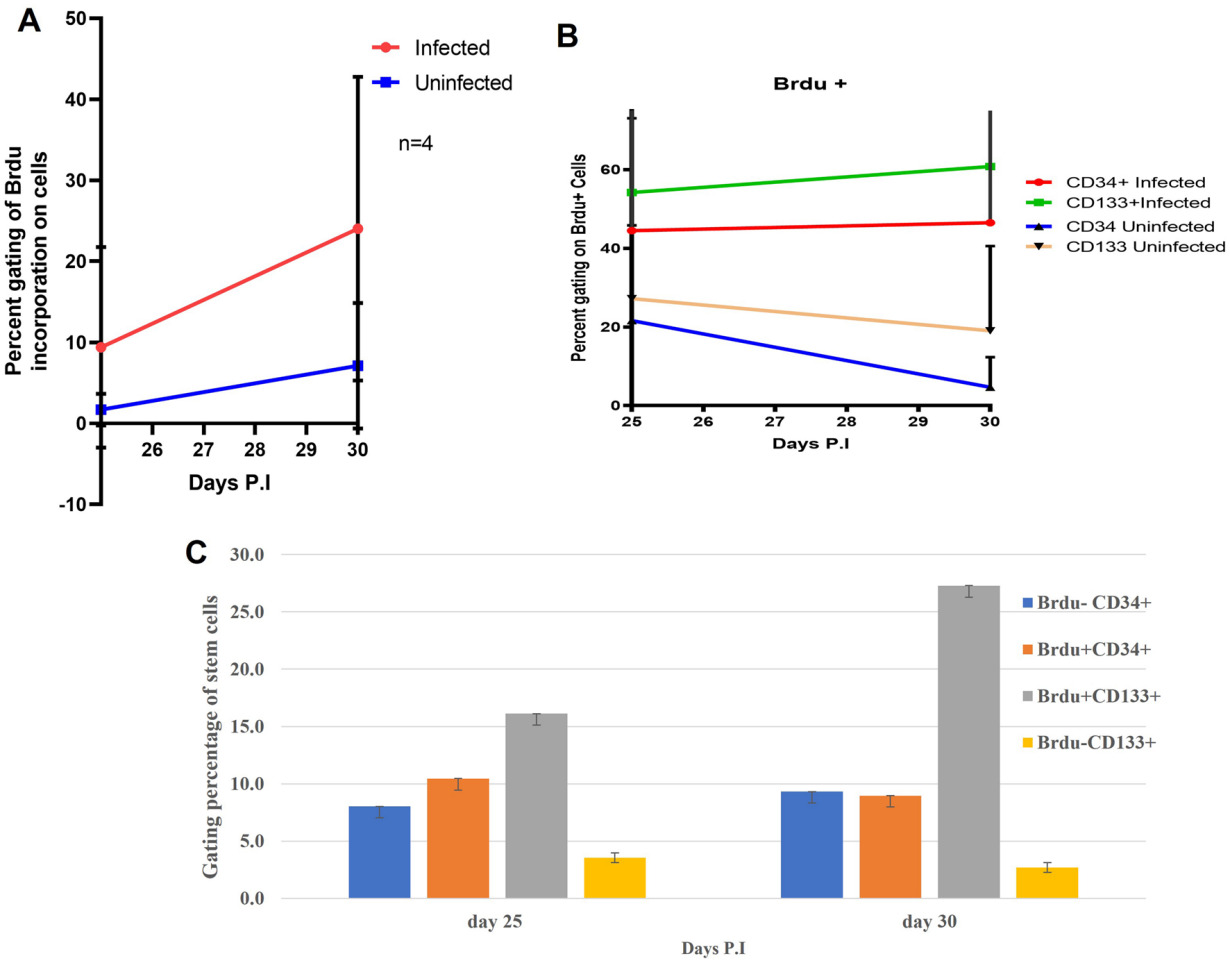
BrdU incorporation into DENV-infected UCB cells. (**A**) Percentage of gated cells of BrdU incorporation in cells. Proliferation in infected cells. (**B**) Percentage of CD133 and CD34 labelled with BrdU+ and BrdU− in DENV-infected cells. The figure shows average data from three infected cells. (**C**) Two days after the incorporation of BrdU, cells were collected on days 27 and 32. The average BrdU incorporation was greatest on day 30, indicating proliferation. The results reflect one representative experiment (out of four).

To understand the process of cell proliferation during prolonged DENV infection in UCBCs, BrdU incorporation was used to study the label-retention ability of prolonged infected cells on days 25 and 30. Multicolor flow cytometry was used based on 10,000 events to determine the percentage of BrdU + CD33 + and BrdU + CD34 + cells. The labeled cells were measured 2 days after BrdU administration, on days 27 and 32. We found that BrdU + CD34 + and BrdU + CD133 + were higher in infected cells compared to uninfected cells (Fig. 4A,B). The decrease in proliferation level after day 25 of BrdU treatment was likely due to the perturbed cell renewal, survival, or differentiation capacity of CD133 and CD34 cells. The high levels of BrdU + CD33 + and BrdU + CD34 + after day 30 of treatment could be correlated with an increased gating percentage of CD34 + /CD133 + NS1 + (Figs. 3B, 4C). Table 1 shows how BrdU uptake kinetics corresponded to lower viral expression on day 25 and higher viral replication on day 30. We assumed that the turnover of quiescent infected cells increased proliferation in DENV-infected UCB. However, we cannot rule out the involvement of other cell types in unfractionated UCB DENV propagation. Whether the amount of released infectious virus produced in unfractionated UCBs was significantly contributed by CD133, CD34, or any other cell types remains to be investigated. Nevertheless, Fig. 4A,B clearly shows that the peak level of BrdU incorporation was higher after day 30 incorporation compared to day 25 in the total cells of UCB. The gating strategy to analyze BrdU incorporation and its gated frequency with CD133 and CD34 is presented in Supplementary Fig. S4.

## Discussion

The prevalence of viral infection in UCB was previously reported^31^, and UCB is known to be rich in HSCs^32,33^. Viruses such as DENV can affect hematopoietic stem cells and were observed to establish infection in tissue cultures. SARS‑CoV‑2 (Coronavirus) was also demonstrated to have the ability to establish an infection in cell culture^34^, but little is known about whether these hematopoietic stem cells are able to harbor the virus. Hence, our aim was to infect UCB with DENV and observe the replication competence in CD133 and CD34 cells. To address whether DENV could establish a persistent infection in these cells, we established an ex vivo model of DENV infection in UCB. In this study, our objective was to explore the idea that CD34 and CD133 are homing sites of persistent DENV infection.

### Persistent infection of DENV with fluctuating virus production after prolonged infection in UCB

When cells are persistently infected with a virus, the yield of the virus gradually decreases^35^. An unexpected outcome in our study was fluctuating virus production for up to a month in the suspension culture of DENV-infected UCB. High, low, and medium virus production was observed. We also observed that the virus disappeared at a certain time point. However, it remains unknown why there was no virus production at certain times, given that DENV was previously shown to have a general trend of a parabolic curve. A more likely scenario is that the virus is released at early time points from the remnants of apoptotic cell death. We suggest that 1MOI virus infecting 1 × 10^6^ cells per ml would equate to 1 × 10^6^ pfu of virus to obtain a true sense of replication kinetics, as a high MOI may attenuate virus replication. However, at the start of infection, virus production was in the 10^3^ range. As a result, a residual amount of 1 × 10^3^ virus or less would be a reasonable quantity to remain simply bound to the cells and become dissociated from cells after a few days. Some DENV-infected UCB cells may have survived the initial infection, resulting in a much slower rate of viral production, as demonstrated by the qPCR with an undetectable and rebounding CT value, as well as the pfu/ml results. We argue that some of the permissive subsets of the cells died, while others controlled the infection or were non-productively infected. Hence, the productive and non-productive phases of dengue virus production indicate that a fraction of the virus particle can stimulate infection. We cannot rule out the possibility that the properties and characteristics of dengue virus particles may change by extending the length of the culture beyond day 14. Moreover, we observed an interesting trend in the induction of IL-6 in DENV-infected HUCB, indicating that cell types in HUCB support DENV infection (Supplementary Fig. S7).

This result also indicates that certain host factors kept the cell alive with an optimal rate of virus production, depending on virus–host interactions. In this culture system, day 20 may represent an eclipse phase of infection, after which the maximum amount of virus is internalized by day 25 and day 30, with viruses produced from these cells having a latent capacity.

The low virus production at later time points below the limit of detection for a plaque assay indicates the presence of defective interfering particles (DI) in the UCB culture setting. DI establishes a persistent infection, thereby interfering with the release of infectious viruses and resulting in low virus titers. We argue that the occurrence of a low number of 1–5 plaques on semi-solid agar of the plaque assay plate equal to 10–100 pfu/ml of virus was due to potentially infectious DENV particles that overcame the mechanism of interference and readily burst after day 20. Above all, the supernatant obtained from DENV-infected UCB stem cells could have a different capacity of infection compared to other cell types. However, this hypothesis requires further investigation.

### Replication competence of DENV in CD133 and CD34 cells in UCB

With respect to long-term virus production from UCBs (tabulated in Table 2), there was an indication that with further incubation of CD133+ and CD34+, production of the virus may be the same. However, the amount of virus produced was considerably lower after day 14 and even undetectable in CD133+ and CD34+, except for UCBC number 4, as seen in Table 2. Moreover, DENV spread out onto the Vero cells from infected CD34+ cells, which is consistent with previous findings^8^. We observed that virus production increased after co-culturing the cells on day 25. This result indicates that cell-free RNA released from prolonged infected cells in the supernatant was inefficient in infecting the cells and non-productive. Analyzing the kinetics of viral spread using CD133 and CD34 in the co-culture potentially indicated that the virus regained its virulence, which could be attributable to cell-to-cell contact. This concept of cells helping cells can mediate the efficient recovery of viruses from specific cells of CD133 and CD34 from their inactive periods. We also demonstrated that the presence of DENV NS1, PrM, capsid, and E proteins in co-cultured infected cells indicated infection onto uninfected Vero cells. This result suggests that HSCs play a role in infection and potentially in reactivating the virus.

Surprisingly, the expression of PrM protein was markedly decreased, while capsid protein was undetectable in CD34+. Our findings are consistent with a previous report that prM content on DENV particles varies^36^. When the co-cultured cells were collected for Western blot on day 7, we observed a decrease in viral load, which could lead to decreased expression of PrM and invisible capsid protein. Moreover, DENV enabled the mis-localization of capsid protein and dysregulated RNA synthesis to facilitate the virus life cycle^37,38^. Additional experiments are required to better understand this phenomenon^8^.

### CD133+/− and CD34+/− with NS1+/− are permissive subsets to maintain long-term infection in UCB

There have been few previous studies on the infection of very primitive HSCs. However, our previous study already showed that infection can persist up to day 14 in HUCB^39^. Interestingly, after a prolonged infection of 4 weeks, there was no discernible difference in the actual number of cells between infected and uninfected cells. Additionally, the infected CD133 and CD34 gating percentage overlapped with uninfected UCB, and, in some instances, it was observed to be higher than that in uninfected cells. At the same time, there was a surge in total cells of DENV-infected UCBCs under ex vivo conditions when fed with CFU-GM. This result suggested that DENV infection could have the ability to activate some degree of proliferation. Cell expansion was also dramatically affected compared to uninfected cells when the cells were fed with CFU-GM at 0.1 ng/ml in the culture medium on days 25 and 30 (Supplementary Fig. S5).

Notably, the observed degree of expression of putative subpopulations of CD133 + NS1+ and CD34 + NS1+, as shown in the t-SNE map, indicates that long-term in vitro culturing entails long-term maintenance of infected cells. It was also noted that despite a prolonged culture duration, CD34 continued to be expressed, unlike CD133 expression, which was undetectable by days 5–10 under confocal microscopy. CD133 is an alternative stem cell marker for CD34 and is expressed in specific CD34 + subpopulations, as well as in CD34−^40^. This marker is known to help maintain hematopoietic systems. It is possible that infection will cause a change that will convert CD133+ to CD34+ and vice versa. The absence of a CD133 signal under confocal microscopy without loss of NS1 in the early phase of infection indicates that CD133 aberrations occurred during active infection. Such infections are reversible following the loss of active viral replication and the inception of quiescent cells, which are slow-dividing cells containing a reservoir of viruses. CD133 is a prominin marker present on diverse kinds of cells such as HSC, endothelial cells, epithelial cells, and various other cell types^41^. However, the precise function of CD133 is unknown. Following viral reactivation after co-culture, the surface protein expression of both CD133 and CD34 was upregulated in co-cultured cells but not on Vero cells. According to our theory in the present study, CD133 and CD34 may serve as intercellular adhesion molecules during infection by DENV, indicating that these cells could aggregate as a homing-associated cell adhesion molecule for attachment of the virus. The increased cell numbers of CD133 and CD34 indicated a mechanism of clonal hematopoiesis, i.e., these proteins underwent self-renewal and produced more HSCs when fighting DENV infection.

### DENV infection modulates the proliferation of CD133 and CD34 cells in UCB

Using BrdU analysis, it was discovered that DENV infection in UCB is primarily associated with both high and low proliferation. Prior studies have demonstrated that chronic infection depletes hematopoietic stem cells^42^. In the present study, we aimed to correlate the cell turnover of CD133 and CD34 with viral production. Interestingly, the difference in BrdU + gated cells between days 25 and 30 was biased towards the differential persistence of DENV and reactivation of the virus (Table 1, Fig. 1A). The attrition of BrdU + gated cells on day 25 suggests that the virus may have constitutively retained the viral gene but without using all the resources of cells to proliferate and remaining in the middle of the productive and non-productive phases of the spectrum. Most importantly, the percentage of BrdU + labeled cells were higher in infected compared to uninfected cells. This result suggests that DENV indeed modulates CD133 and CD34 cells in UCB. These results from four independent donors were reproducible. According to our interpretation, cells carrying reactivable viruses can halt proliferation, thereby causing the cells to arrest themselves to limit proliferation and keeping the infected cells safe to resume infection.

## Conclusions

The majority of DENV is subclinical, and classical dengue subsides frequently. One of the many factors influencing DENV pathogenesis and spread is virus transmissibility^43^**.** There are many missing pieces of information regarding DENV entry, although cell-free virus entry is not efficient for productive infection. The mechanism of cell–cell transmission for virus egress will be beneficial in understanding persistent infection in cell cultures. Collectively, these findings indicate that DENV could have long-term consequences for the hematopoietic niche in cord blood settings. Since DENV has four serotypes, and memory cells provide only partial immunity to four DENV serotypes, it is conceivable that primitive HSCs could behave as decoys to control virus replication and as a strategy for infection. Conclusively, our investigation may be crucial for a future line of study in DENV transmissibility by primitive HSCs as reflected by their ability to retrieve replication-competent virus. Supposedly, reactivating the virus from its quiescent phase can maximize the virus’s opportunities to mutate and develop a new variant. This factor impedes the control of DENV transmission.

## Supplementary Information

Supplementary Information.

## Data Availability

All data generated or analyzed during this study are included in this published article and its supplementary information file.
