# Supplementary material for: The CD133 and CD34 cell types in human umbilical cord blood have the capacity to produce infectious dengue virus particles
Source: Sci Rep. 2023 Jun 29;13:10513. doi: 10.1038/s41598-023-37707-8 (PMC10310799; doi:10.1038/s41598-023-37707-8)
Supplement: Supplementary file 1 — Supplementary Information. [file 41598_2023_37707_MOESM1_ESM.pdf]

Supplementary Figures

The CD133 and CD34 cell types in human umbilical cord blood have the capacity to produce infectious dengue virus particles

Amrita Vats[1], Tzu-Chuan Ho[1], Irwin Puc[1], Chiung-Hsin Chang[3], Guey. Chuen Perng [1,2], Po-Lin Chen[4]

A

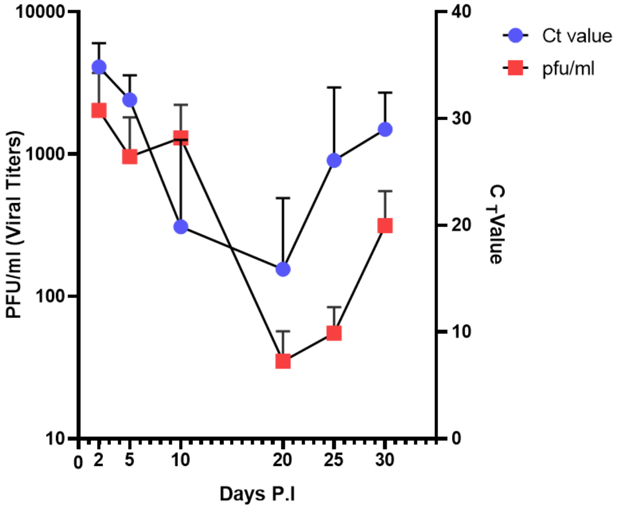

B

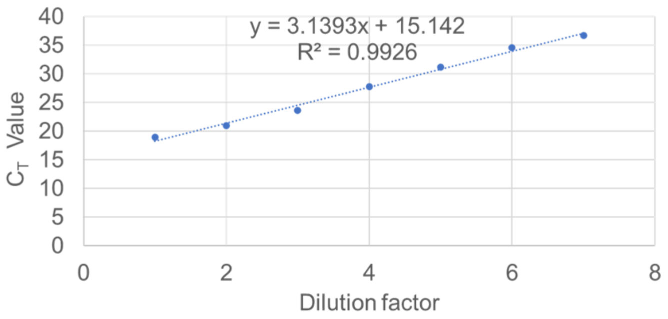

Supplementary Figure S1: Comparison of threshold cycle and pfu/ml in DENV infected UCB culture supernatant. (A) Quantitative real time qPCR to determine concentration of RNA. The C_T value for each DENV infected UCB supernatant at indicated time were determined after converting to cDNA using super script III first-strand synthesis system for RT-PCR and random hexamers. The graph represents comparison of pfu/ml and C_T value from UCB supernatant (n=5). The Unpaired-T test was applied for statistical correlation. **(B)** A six-fold dilution of stock virus (10^7 PFU/ML) was used to generate the standard curv

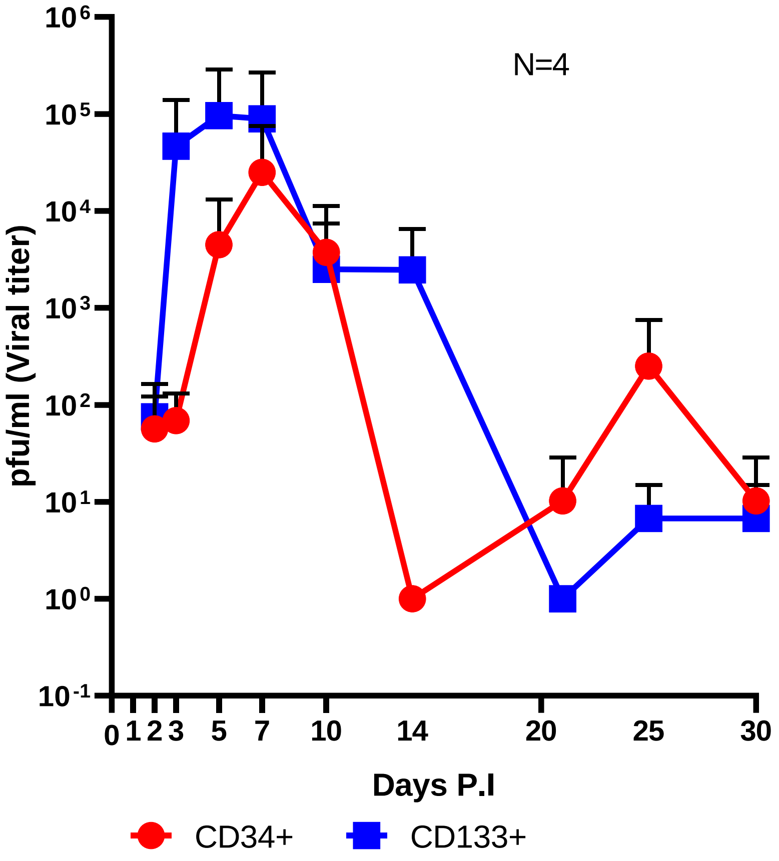

Supplementary Figure S2. Confirmation of the infectivity of virus in specific sorted CD133+ and CD34+ cells Viral load from the supernatant in $CD133^+$ and $CD34^+$ cells(n=4). High viral particle was produced by DENV infected $CD133^+$ and $CD34^+$ cells. The DENV infected $CD34^+$ cells did not shed the virus at day 14 .$CD133^+$ and $CD34^+$ reactivated the viral particle after a silent phase at day 20.

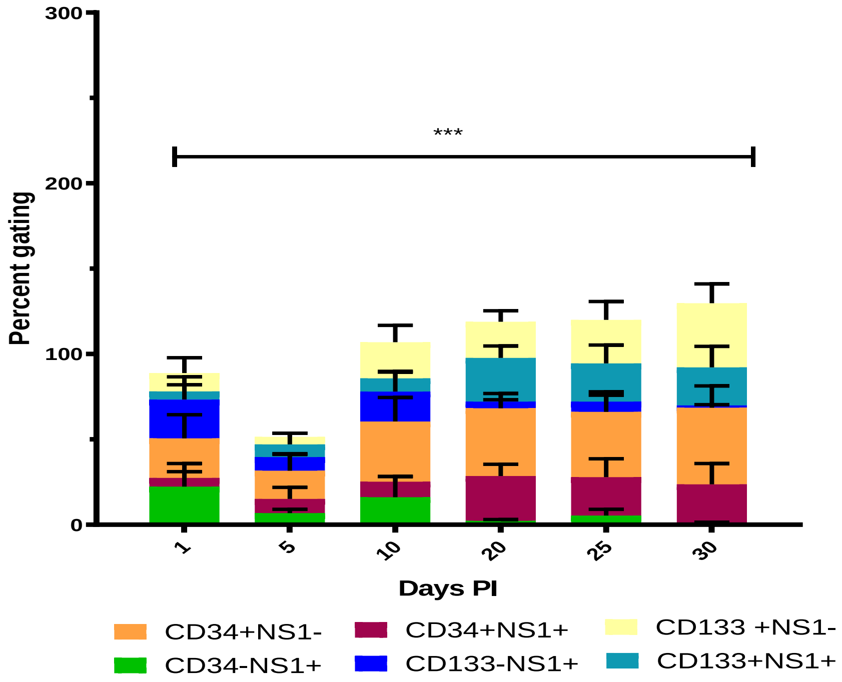

Supplementary Figure S3: Expression of CD133, CD34 representing percent of gated cells of CD133 and CD34 in infected and uninfected UCB cells. The Ordinary one-way Anova test was applied for statistical analysis (p=**0.0021)

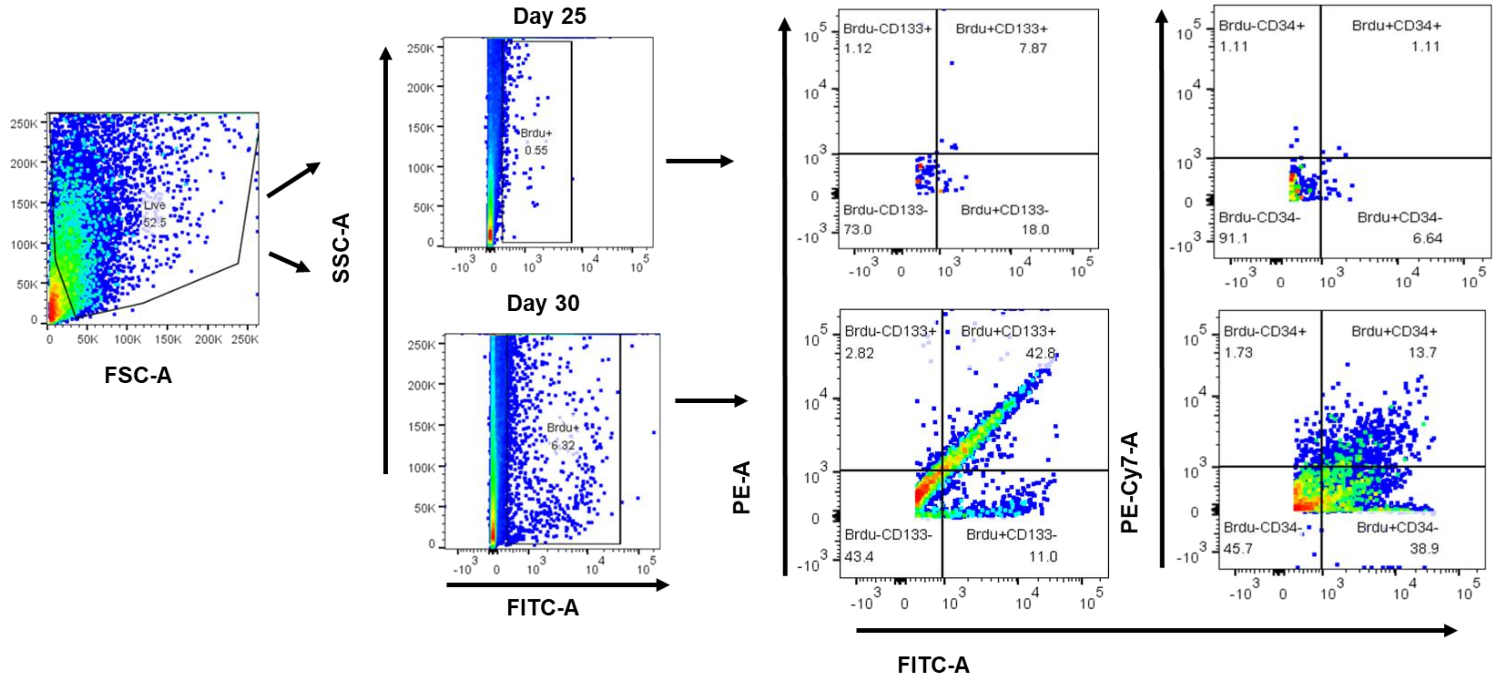

Supplementary Figure S4: Gating strategy to analyzed BrdU incorporation and its gated frequency with CD133 and CD34

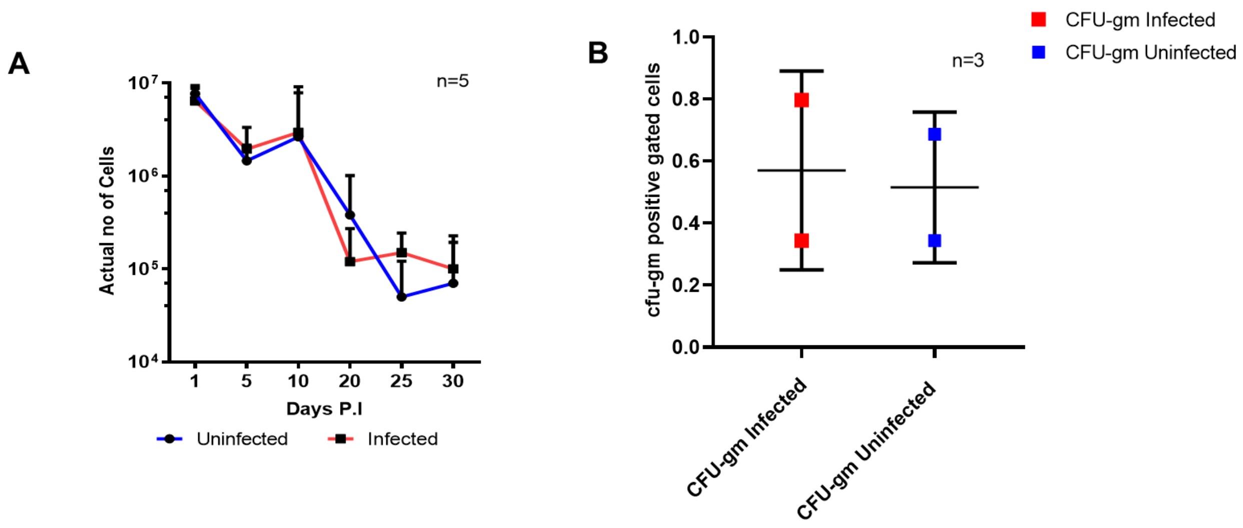

Supplementary Figure S5: **(A)** Cell viability and actual number of cells in infected and uninfected at time interval of 30 days. 500 µl of medium was exchanged at intervals of 2, 5,10 days at 30-day intervals (N=5). **(B)** Cell expansion when fed with cfu-gm at (0.1ng/ml) in the culture medium at day 25 and day 30 in DENV infected and uninfected cells. The cells for the cfu-gm positive gated cells were analyzed after staining with CD133 and CD34 and NS1 surface marker using flow cytometry.

Raw data

Figure 2A **-Viral load after post-co-culture**

CD34+

Days P. I	Donor 1	Donor 2	Donor 3	Donor 4
1				
2	50	25	50	25
3	250	25	375	50
5	1	250	1	1
7	1	50	1	1

CD133+

Days P. I	Donor 1	Donor 2	Donor 3	Donor 4	Donor 5
1			1	1	
2	125	50	125	125	1300
3	12.5	1	50	12.5	19250
5	1	1	1	1	87500
7	1	1	1	1	875

***1 represents no virus in the plaque assay.**

Figure 2C: Gated percentage of stem cells in co-cultured

	CD34	CD133	Vero CD34	Vero CD133
Number of gated cells	7.32	19.75	0.24	0.4
	0.22	3.15	1.9	3.28

Figure 3A: Gating percentage of CD133 and CD34+

Infected CD133+

Days P. I	Donor 1	Donor 2	Donor 3	Donor 4	Donor 5	Donor 6	Donor 7	Donor 8	Donor 9	Donor 10	Donor 11
1	0.19	0.33	3.52	0.03	35.53	0.65	45.17	3.52	1.28	24.53	4.89
5	0.24	4.96	4.61	1.77	16.67	2.06	37	4.61	0.2	1.61	8.39
10	4.3	1.44	7.31	0.57	18.19	19.7	52	7.31	31.18	1.39	0.22
20	2.95	0.82	17.03	3.37	7.03	0.15	0.37	23.27	22.78	0.18	0.2
25	0.31	18.56	25.74	0.27	20.97	1.28	0.73	25.09	29.96	0.51	0.2
30	0.4	0.66	0.9	1.42	28.44	2.93	1.44	0.95	0.47	0.19	0.35

Uninfected CD133+

Days P.I	Donor 1	Donor 2	Donor 3	Donor 4	Donor 5	Donor 6	Donor 7	Donor 8	Donor 9	Donor 10	Donor 11
1	0.24	0.2	5.53	0.15	58.76	1.13	49.32	5.53	0.09	17.78	16.59
5	0.07	3.46	3.35	0.17	63.91	3.23	26.05	3.35	4.05	0.78	4.58
10	5.45	0.15	4.49	1.13	0.47	0.76	1.02	4.49	3.28	0.08	0.2
20	6.31	0.42	8.94	1.66	2.45	0.72	0.61	8.94	7.67	16.59	2.51
25	0.58	12.51	4.93	0.73	9.22	6.3	0.16	4.93	0.88	0.85	0.69
30	0.36	0.35	1.08	6.44	8.16	1.98	22.71	1.08	0.02	0.18	1.96

Infected CD34+

Days P. I	Donor 1	Donor 2	Donor 3	Donor 4	Donor 5	Donor 6	Donor 7	Donor 8	Donor 9	Donor 10	Donor 11
1	0.45	0.68	3.91	0.48	32.05	1.04	42.13	1.03	1.17	20.15	2.72
5	0.05	3.62	1.95	0.92	9.1	0.73	12.98	23.17	0.29	0.23	0.63
10	4.47	1.92	4.95	0.65	15.12	10.11	0.48	3.96	34.19	2.02	1.02
20	1.62	1.21	23.27	3.72	9.36	0.03	0.3	2.13	17.37	0.46	1.87
25	0	21.97	25.09	0.49	17.13	0.64	0.61	5.03	23.91	0.13	1.01
30	1.28	0.22	0.95	1.06	18.92	0.9	2.75	0.37	0.4	2.26	0.84

Uninfected CD34+

Days P. I	Donor 1	Donor 2	Donor 3	Donor 4	Donor 5	Donor 6	Donor 7	Donor 8	Donor 9	Donor 10	Donor 11
1	0.08	0	5.77	0.19	71.2	1.12	50.28	3.07	0.15	9.13	4.02
5	0.11	1.09	2.12	0.16	30.12	0.46	18.06	0.84	3.35	0.54	6.21
10	0.99	0.1	5.94	1.41	0.19	1.5	2.35	7.69	2.55	0.41	0.16
20	4.02	0.14	13.07	2.41	1.41	0.51	0	5.59	6.58	9.91	4.39
25	1.16	16.14	1.03	2.79	4.83	8.62	0.12	0.31	0.96	0.93	0.41
30	0.45	0.2	1.01	5.74	4.5	1.16	20.34	0.08	0.12	0.68	1.92

Figure 3B Gating percentage of NS1+ with CD133 and D34

CD34-NS1+

Days PI	Donor 1	Donor 2	Donor 3	Donor 4	Donor 5
**1**	0.21	1.31	31.7	6.7	71.6
**5**	6	0.38	6.61	6.86	14.2
**10**	0.4	0.79	15.3	0.82	63.3
**20**	0.74	2.1	3.25	0.31	4.83
**25**	0.24	0.87	19.1	0.08	6.47
**30**	0.15	0.46	1.7	0.48	2.44

CD34+NS1+

Days PI	Donor 1	Donor 2	Donor 3	Donor 4	Donor 5
**1**	19.5	1.98	0.12	3.74	0.59
**5**	35.1	0.78	0.28	5.19	0.19
**10**	20.46	8.99	2.5	8.54	4.92
**20**	34.2	46.6	20.8	5.53	24.4
**25**	60.6	31.9	1.84	9.71	8.77
**30**	20.1	15.6	6.08	69.9	1.22

CD34+NS1-

Days PI	Donor 1	Donor 2	Donor 3	Donor 4	Donor 5
**1**	69	41.3	0.061	5.18	0.23
**5**	15.5	52.4	0.81	13.8	0.062
**10**	58	42	2.15	71.2	3.06
**20**	59.5	47.9	20.9	53	17.1
**25**	39.1	64.8	13.1	64	9.83
**30**	77.2	64.8	48.8	27.8	6.34

CD133-NS1+

Days PI	Donor 1	Donor 2	Donor 3	Donor 4	Donor 5
1	0.52	3.84	29.5	8.2	71.6
5	9.17	0.47	8.06	8.95	13.2
10	0.68	0.99	17.6	4.73	63.3
20	3.34	4.05	4.11	0.44	7.52
25	0.32	1.57	19.8	0.51	8.02
30	0.15	0.84	2.55	0.75	2.21

CD133+NS1+

Days PI	Donor 1	Donor 2	Donor 3	Donor 4	Donor 5
1	20.3	1.09	0.35	1.36	0.54
5	33.5	0.89	0.23	2.48	0.085
10	21.4	7.92	1.1	6.44	2.01
20	34.2	46.7	19	5.4	22.7
25	59.7	33.3	1.08	9.27	8.41
30	20.1	14.1	6.67	69.7	0.87

CD133+NS1-

Days PI	Donor 1	Donor 2	Donor 3	Donor 4	Donor 5
1	46	7.11	0.076	0.64	0.11
5	9.62	9.49	0.36	2.64	0.028
10	52.4	20.4	0.63	32.3	0.46
20	19.7	45.8	15.5	15.7	9.23
25	34.3	61.7	4.99	23.8	3.13
30	69.3	53.5	34.8	27.3	2.99

Figure 4A Brdu proliferation assay

Brdu- CD34+				
Days P.I	Donor 1	Donor 2	Donor 3	Average
25	1.11	2.03	20.9	**8.0**
30	1.73	0.53	25.7	**9.3**

Brdu+CD34+				
Days P. I	Donor 1	Donor 2	Donor 3	Average
25	1.11	30.2	0.068	**10**
30	13.7	13.1	0.094	**9**

Brdu+CD133+				
Days P. I	Donor 1	Donor 2	Donor 3	Average
25	7.87	40.4	0.057	**16**
30	42.8	38.9	0.15	**27**

Brdu-CD133+				
Days P.I	Donor 1	Donor 2	Donor 3	Average
25	1.12	6.95	2.59	**3.6**
30	2.82	0.85	4.41	**2.7**

Figure 4B

CD34+Infected				
Days P.I	Donor 1	Donor 2	Donor 3	Donor 4
25	91	0	19.4	67.6
30	95.2	21.7	42.6	26.7

CD34 Uninfected				
Days P.I	Donor 1	Donor 2	Donor 3	Donor 4
25	41.7	0	1.33	43.5
30	0	16	0	2.68

Figure 4B
CD133+Infected

Days P.I	Donor 1	Donor 2	Donor 3	Donor 4
**25**	99	0	38.7	79.2
**30**	98.2	45.4	56.8	43

CD133 Uninfected

Days P.I	Donor 1	Donor 2	Donor 3	Donor 4
**25**	95.7	0	2.3	10.9
**30**	3	28	0	45.3

Figure 4C

Brdu- CD34+

Days P. I	Donor 1	Donor 2	Donor 3	Average
**25**	1.11	2.03	20.9	**8.0**
**30**	1.73	0.53	25.7	**9.3**

Brdu+CD34+

	Donor 1	Donor 2	Donor 3	Average
**25**	1.11	30.2	0.068	**10**
**30**	13.7	13.1	0.094	**9**

Brdu+CD133+

	Donor 1	Donor 2	Donor 3	Average
**25**	7.87	40.4	0.057	**27**
**30**	42.8	38.9	0.15	

Brdu-CD133+

	Donor 1	Donor 2	Donor 3	Average
**25**	1.12	6.95	2.59	**3.6**
**30**	2.82	0.85	4.41	**2.7**

Supplementary Figure S5 A No of cells remain viable

Uninfected

Days P. I	Donor 1	Donor 2	Donor 3	Donor 4	Donor 5
1	9900000	7900000	8400000	5300000	7000000
5	1800000	1700000	2300000	850000	650000
10	12000000	400000	350000	300000	130000
20	50000	1500000	150000	0	200000
25	150000	0	0	0	100000
30	0	0	350000	0	0

Infected

Days P. I	Donor 1	Donor 2	Donor 3	Donor 4	Donor 5
1	7500000	8900000	3700000	4100000	8000000
5	3900000	1900000	1500000	50000	2400000
10	14000000	400000	250000	0	100000
20	350000	200000	0	0	50000
25	250000	150000	0	150000	200000
30	100000	50000	100000	0	250000

Supplementary Figure S5B Number of gated cells of CD133+CD34+NS1+ subsets

CFU-gm Infected

Days P. I	Donor 1	Donor 2	Donor 3
**25**	1	0.01	0.02
**30**	0.42	1.94	0.03

cfu-gm uninfected

Days P. I	Donor 1	Donor 2	Donor 3
**25**	1	0.01	0.02
**30**	2	0.01	0.05

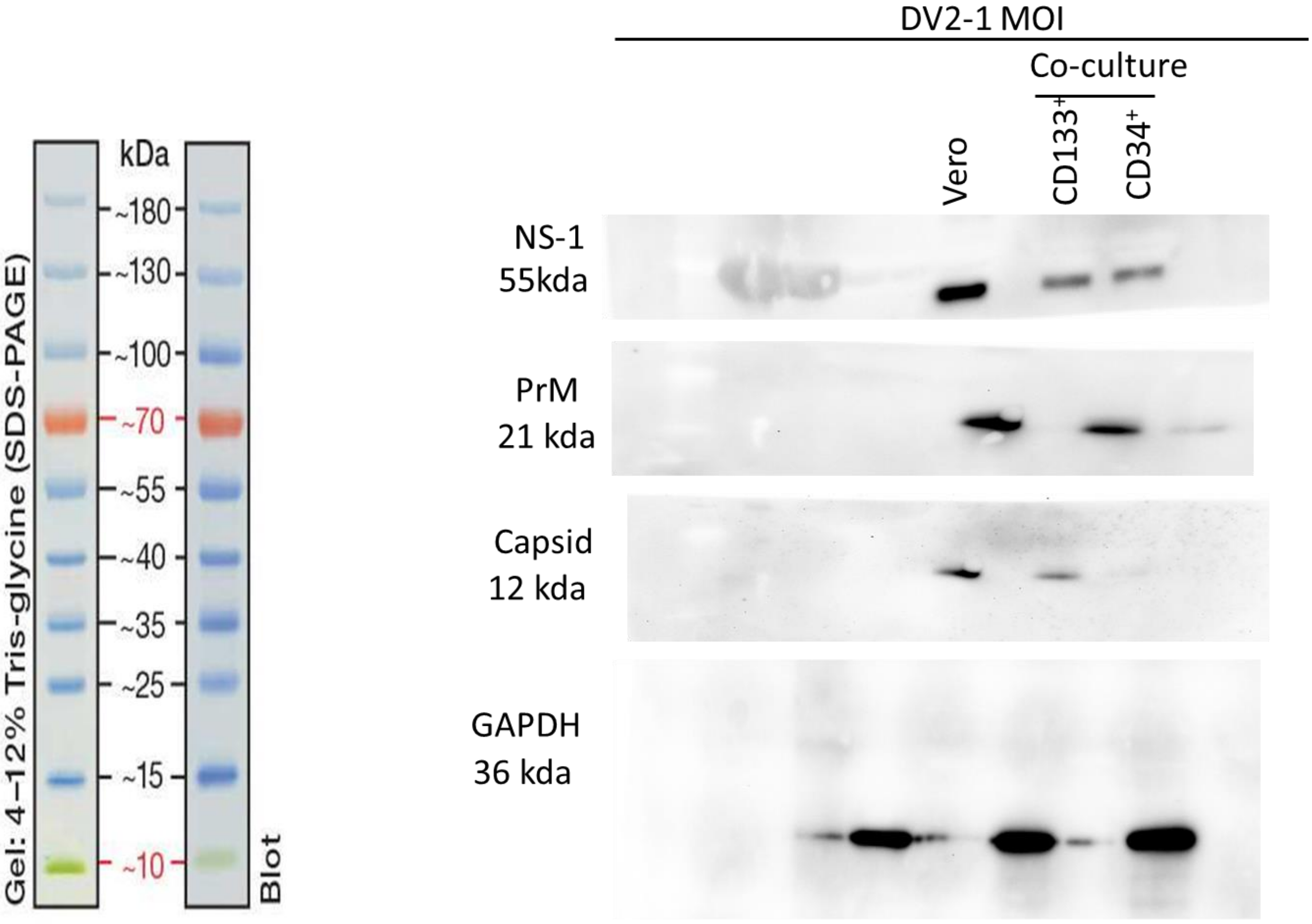

Supplementary Figure S6A: Original blot images of NS1, PrM, Capsid and GAPDH Figure 2 B. Using a marking scale vertically, the blots were precisely cut from the desired molecular weight protein ladder, prior to hybridization with respective antibodies.

DV2 MOI= 1

Vero cells CD34 CD133 CD45

E protein

55kda

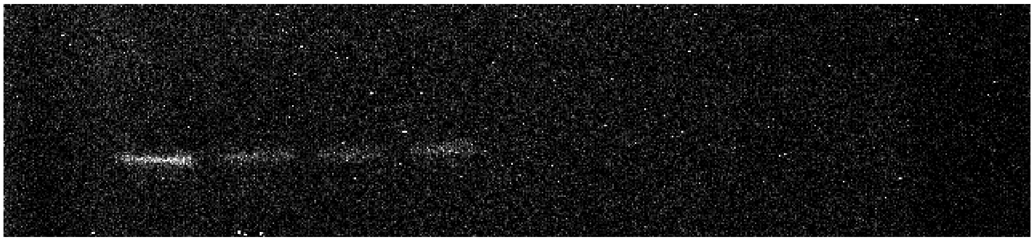

CD34 CD133

E protein

55kda

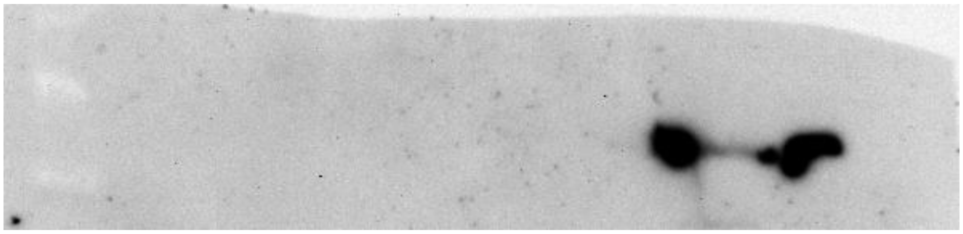

Supplementary Figure S6B: An additional image of E protein from replicates 3 and 4 of DENV-infected UCB co-cultured cells, to confirm that DENV E protein was present in DENV-infected co-cultured cells after day 7 of infection. For Figure 2B**:** Using a marking scale vertically, the blots were precisely cut from the desired molecular weight protein ladder, prior to hybridization with respective antibodies.

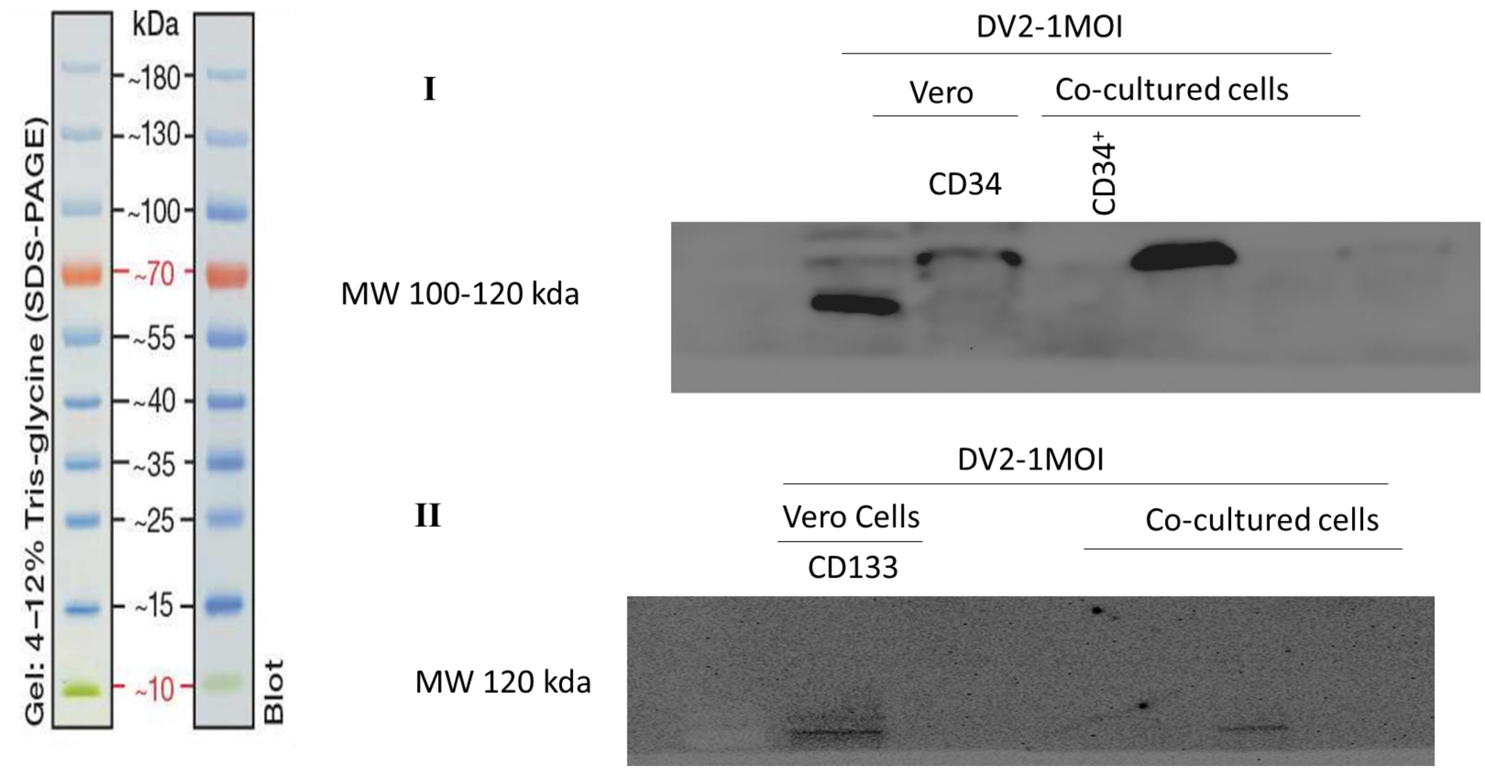

Supplementary Figure S6C: I. Original uncropped blot images of DENV infected Vero cells and co-cultured CD34 protein shown in Figure 2B. II.The blot images of CD133 protein and GAPDH which were performed on another replicate of DENV-infected UCB co-cultured cells, were provided to confirm that CD133 protein was present in DENV-infected co-cultured cells after day 7 of infection. Additional image for Figure 2D. Using a marking scale vertically, the blots were precisely cut from the desired molecular weight protein ladder, prior to hybridization with respective antibodies.

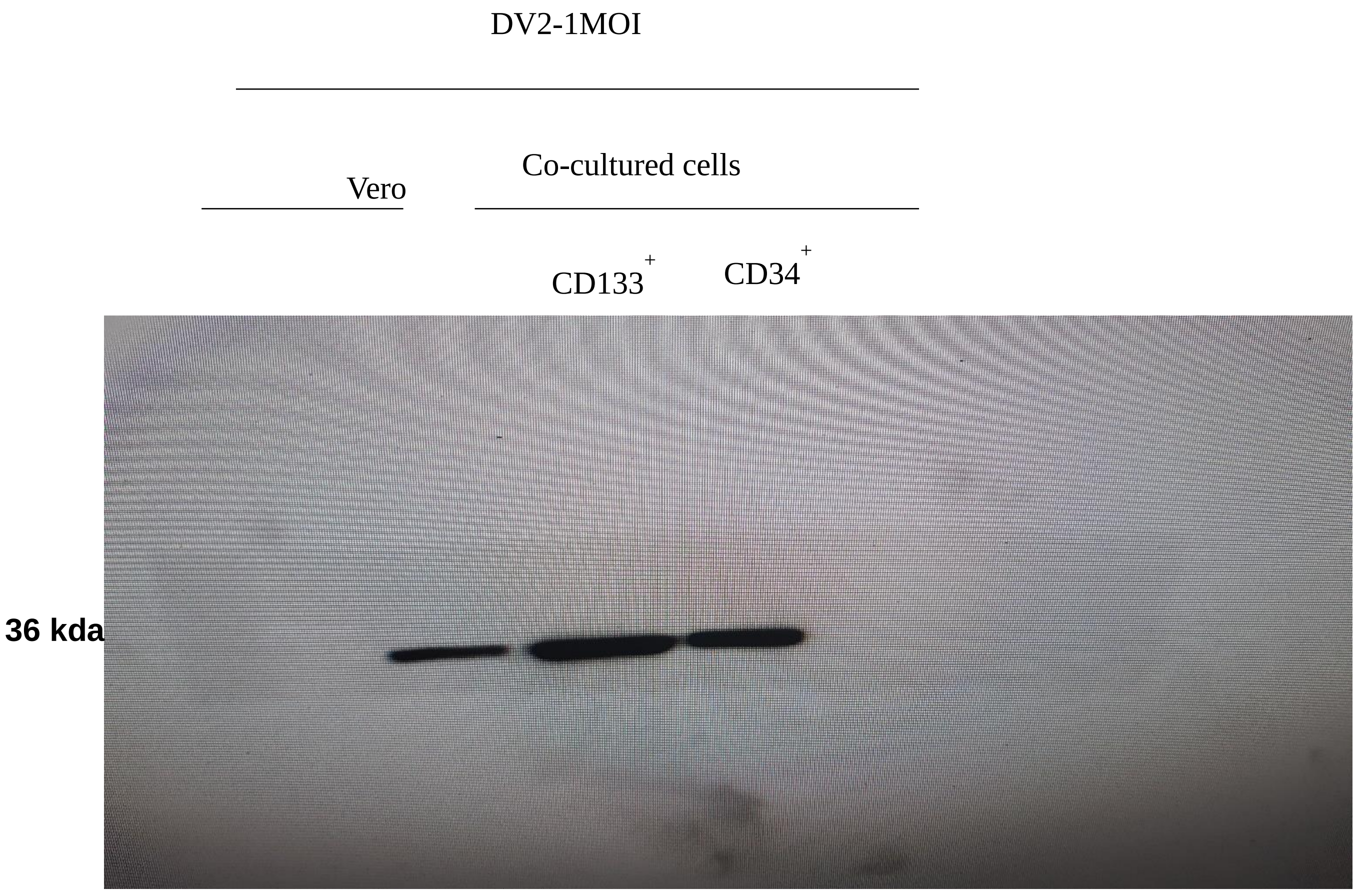

Supplementary Figure S6D: Original uncropped full length blot image of GAPDH. The image provided here was performed on another set of DENV-infected UCB co-cultured cells, was present in DENV-infected co-cultured cells after day 7 of infection. For Figure 2D

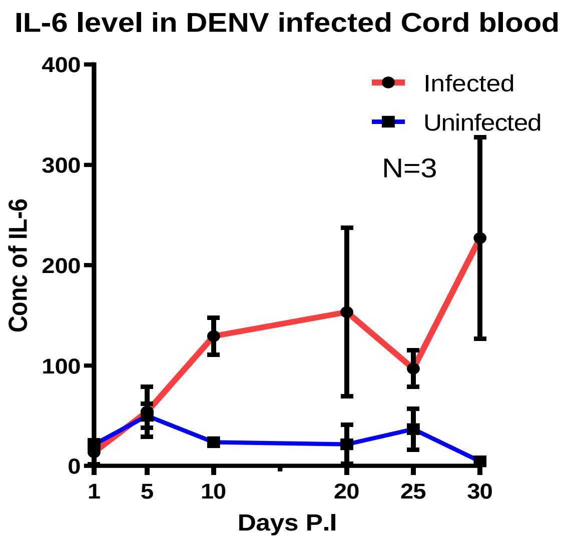

Figure S7: Production of IL-6 in DENV infected HUCB

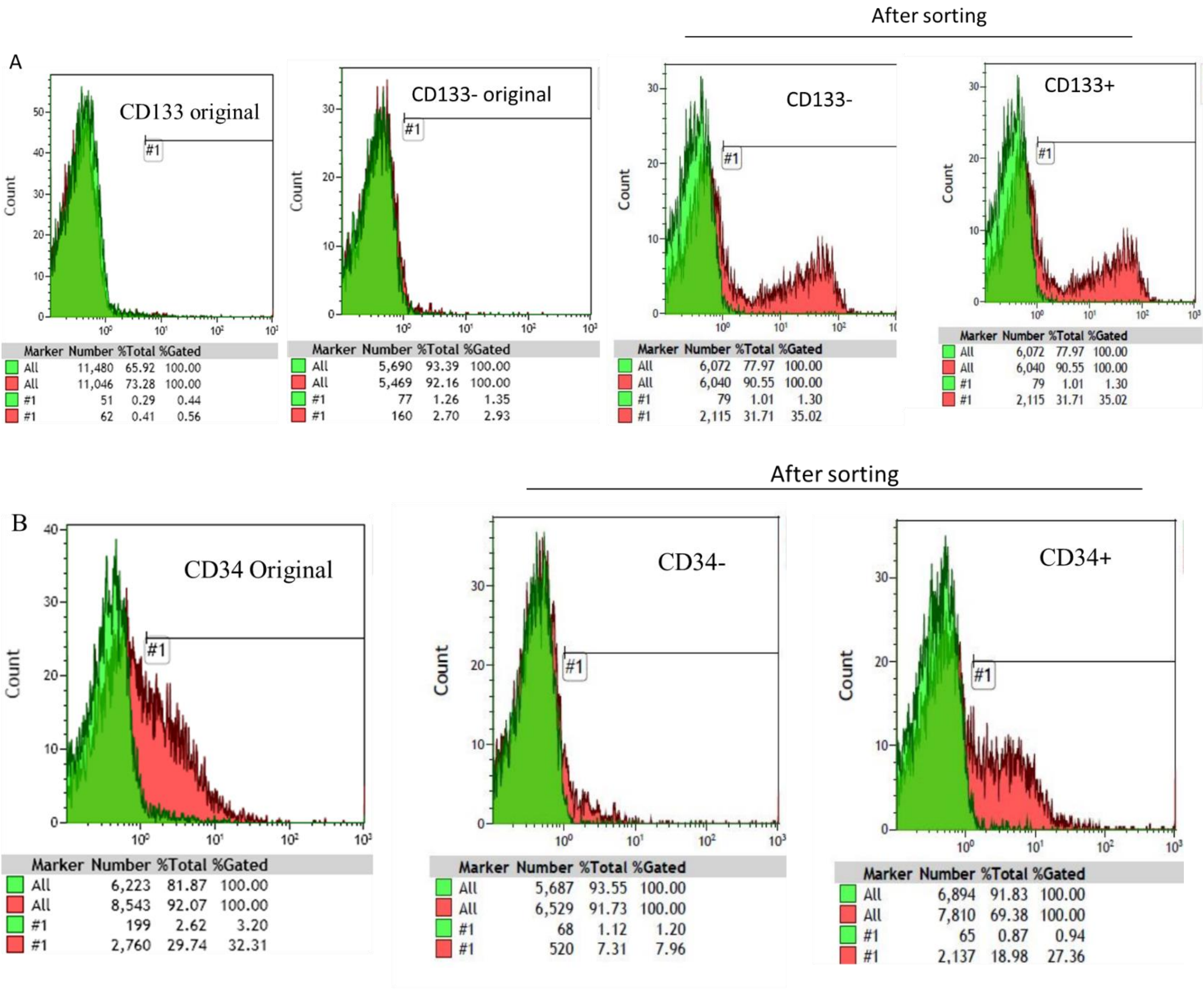

Figure S8: Purity of CD133 and CD34 obtained from HUCB: Purity of the gating percentage of $CD133^{+}$ and $CD34^{+}$ cells obtained after magnetic bead sorting using flow cytometry from one of the representative HUCB donor.
